# The Polymorphism in* ADORA3* Decreases Transcriptional Activity and Influences the Chronic Heart Failure Risk in the Chinese

**DOI:** 10.1155/2018/4969385

**Published:** 2018-05-31

**Authors:** Hai-Rong He, Yuan-Jie Li, Gong-Hao He, Hua Qiang, Ya-Jing Zhai, Mao Ma, Ya-Jun Wang, Yan Wang, Xiao-Wei Zheng, Ya-Lin Dong, Jun Lyu

**Affiliations:** ^1^Clinical Research Center, The First Affiliated Hospital of Xi'an Jiaotong University, Xi'an 710061, China; ^2^Department of Human Anatomy, Histology and Embryology, Medical College, Xi'an Jiaotong University, Xi'an 710061, China; ^3^Department of Pharmacy, Kunming General Hospital of Chengdu Military Region, Kunming 650032, China; ^4^Department of Cardiovascular Medicine, The First Affiliated Hospital of Xi'an Jiaotong University, Xi'an 710061, China; ^5^Department of Pharmacy, The First Affiliated Hospital of Xi'an Jiaotong University, Xi'an 710061, China; ^6^Physical Examination Department, The First Affiliated Hospital of Xi'an Jiaotong University, Xi'an 710061, China

## Abstract

**Aim:**

To investigate the genetic contribution of adenosine A3 receptor (ADORA3) gene polymorphisms in the pathogenesis of chronic heart failure (CHF).

**Methods:**

Firstly, a case-control study was performed to investigate the association of ADORA3 polymorphisms with CHF risk. Three hundred northern Chinese Han CHF patients and 400 ethnicity-matched healthy controls were included. Four polymorphisms were genotyped. This case-control study was also replicated in 304 CHF patients and 402 controls from southern China. Finally, the functional variability of positive polymorphism was analyzed using luciferase reporter assay and real-time PCR.

**Results:**

Overall, the rs1544223 was significantly associated with CHF risk under the dominant model (*P* = 0.046, OR = 1.662, 95% CI = 1.009–2.738). But it did not affect disease severity. These results were also consistent in replicated population. In addition, the transcriptional activity for promoter with the A allele was lower than that with the G allele (*n* = 3, 4.501 ± 0.308 versus 0.571 ± 0.114, *P* < 0.01) and ADORA3 mRNA levels were significantly higher in GG homozygotes than subjects carrying GA (*n* = 6, 0.058 ± 0.01 versus 0.143 ± 0.068, *P* = 0.004) or AA genotypes (*n* = 6, 0.065 ± 0.01 versus 0.143 ± 0.068, *P* = 0.008).

**Conclusions:**

Should the findings be validated by further studies with larger patient samples and in different ethnicities, they may provide novel insight into the pathogenesis of CHF.

## 1. Introduction

Chronic heart failure (CHF) has a high prevalence in older individuals and is a major cause of morbidity, mortality, hospitalizations, and disability [1]. Despite recent advances in the treatment of heart failure, it remains a condition with significant morbidity and mortality, with a 5-year mortality rate that rivals most cancers [2]. The rate of mortality among patients with CHF after hospital admission or within one year after admission is reported to be 40% [3, 4]. The search for new effective drug targets and prevention strategies has thus become a serious challenge in the clinical setting.

Numerous physiological processes are modulated by adenosine (Ado), which is an endogenous and cardioprotective nucleoside [5, 6]. There is experimental evidence to show that endogenous Ado improves the rate of oxygen utilization by optimizing the relationship between myocardial energy production and consumption [7, 8]. In addition, Ado has recently been shown to improve cardiac systolic and diastolic function by reducing end-diastolic pressure and increasing the rate of left ventricular pressure reduction [9]. It also has electrophysiological effects that induce a reduction in heart rate [10] and is thought to exert a protective effect on aging hearts due to its antiinflammatory, antiapoptotic, antiadrenergic, and antioxidant effects [11]. Furthermore, this nucleoside can protect the heart from ischemia–reperfusion injury and cardiac remodeling, thus preventing the progress of heart failure [12].

Ado mediates its various cardiovascular actions via four known receptor subtypes: A_1_, A_2A_, A_2B_, and A_3_. Some studies have demonstrated the role of the Ado A_3_ receptor* (ADORA3)* on the heart. It is thought that* ADORA3* signaling protects cardiomyocytes against the damage caused by ischemia via short-term preservation by ATP, as well as protecting them from contractile dysfunction and energy depletion by normalizing intracellular Ca^2+^. This cardioprotective effect has been observed after induced ischemia in experimental mouse and rabbit animal models [13–17], and following the administration of doxorubicin in rats [18]. Ashton et al. also found that reduced A3 adenosine receptor transcription may contribute to impaired ischemic tolerance in aged mouse hearts [19]. In addition,* ADORA3* activation also induces myocardial cells apoptosis [20]. Del et al. further demonstrated its influence on heart failure with the finding that* ADORA3* mRNA expression in the left ventricle of failing minipig hearts was significantly higher than that in control healthy minipig hearts [21].

The activity of* ADORA3* is an important factor that affects the cardioprotective properties of Ado. The activity of* ADORA3* in vivo could be affected by gene polymorphisms. It is thus reasonable to hypothesize that single nucleotide polymorphisms (SNPs) in the gene encoding* ADORA3* influence its activity and further affect CHF susceptibility and progress—to the best of our knowledge this issue has not been investigated previously. This hypothesis was tested by establishing the relationship between certain tag-SNPs of* ADORA3* and susceptibility to CHF among a Chinese Han population and demonstrating the functional contribution of genetic polymorphisms of the ADORA3 gene, to provide insights into the prevention and individualized treatment of CHF.

## 2. Materials and Methods

### 2.1. The Study Population

Chinese CHF patients (age > 18 years old) were recruited from the First Affiliated Hospital, College of Medicine, Xi'an Jiaotong University (between 2013 and 2014), and the Kunming General Hospital of Chengdu Military Region (from 2012 to 2014). The inclusion criterion was diagnosis of heart failure according to the Guidelines for Diagnosis and Treatment of Heart Failure in China (2013). The cause of heart failure was determined in each patient by clinical assessment and echocardiography. The patient exclusion criteria included the following: presence of severe hepatic or renal insufficiency, tumors or malignant disease, acute attack of CHF, severe acute infection, metabolic disorders, and acute myocardial infarction (MI) without revascularization within 2 weeks. All sample collection was completed according to strict inclusion criteria by two visiting staffs and were reviewed by the Chief Physician of Cardiology. Ultimately, 300 patients from Xi'an and 304 patients from Kunming were eligible for inclusion in this study.

The control group comprised sex- and ethnicity-matched healthy volunteers from the Medical Examination Center of the same hospital (age > 18 years) who had no known personal or family history of cardiovascular disease. A detailed interview was used to obtain information about personal and familial history in the framework of a physical examination by expert physicians to identify symptom-free subjects and to exclude those who were suspected of having any form of vascular disease. Finally, 400 controls from Xi'an and 402 controls from Kunming were included in this study.

Demographic and clinical characteristics (including gender, age, and traditional cardiovascular risk factors) were obtained from interviewer-administered health-risk questionnaires. The subjects were considered to have hypertension according to the Guidelines for Prevention and Treatment of Hypertension in China (2013) or if they were taking antihypertensive drugs. Dyslipidemia was defined according to the Guidelines on Prevention and Treatment of Blood Lipid Abnormality in Chinese Adults (2007), and diabetes was defined in agreement with the Guidelines for Prevention and Treatment of Diabetes in China (2013). Information regarding smoking status was self-reported. Individuals were designated “smokers” if they have reported that they had smoked at least five cigarettes daily for at least the past 12 months [22].

### 2.2. Genotyping Assay

The blood samples were collected into tubes containing ethylenediaminetetraacetic acid. After centrifugation, the samples were stored at −80°C until analysis. The standard phenol–chloroform extraction method was used to extract genomic DNA from whole blood. DNA concentration was measured by spectrometry (DU530 UV/VIS spectrophotometer, Beckman Instruments, Fullerton, CA, USA). Three tag-SNPs (i.e., rs1415792, rs1544223, and rs3393), which captured the majority of known common variations of* ADORA3* according to Chinese Han population data from HapMap (http://www.hapmap.org), and one SNP (rs35511654) that was yielded by literature searched were selected for the present study. Sequenom MassARRAY Assay Design 3.0 Software was used to design Multiplexed SNP MassEXTEND assay [23–25]. SNP genotyping was performed using the Sequenom MassARRAY RS1000 system according to the standard protocol recommended by the manufacturer [23]. The corresponding primers used for each SNP in the present study are listed in Supplementary Table S1. Sequenom Type 4.0 Software was used to perform data management and analyses [23–26].

### 2.3. Luciferase Assay

The rs1544223 allelic differences in promoter activity in the human HEK293T line were confirmed using the pGM-Lu vector. Cells were transfected with these reporter constructs and with pRL-TK* renilla* (Promega) luciferase vector as a normalization control. We constructed HEK293T cells (5 × 10^5^ per Petri dish) which were cotransfected with 1.8 *μ*g pGM-Lu-SNP (firefly luciferase) and 0.2 *μ*g pRL-TK (renilla luciferase) (Promega) plasmid using 5 *μ*l Lipofectamine™ 2000. After transfection, cells were lysed 72 h in 250 *μ*l 1x Passive Lysis Buffer and luciferase activity was measured with fluorescence microscope (Olympus) according to Dual Luciferase Reporter Gene Assay Kit (Promega). Relative luciferase activity of ADORA3 reporter constructs was calculated as the ratio of firefly luciferase activity to renilla luciferase. Reporter assays shown were the average of three parallel test results.

### 2.4. Real-Time PCR Analysis

Blood samples were collected by Mr. Wang, the director of Medical Examination Center, from 107 healthy volunteers. The rs1544223 genotypes of these samples were performed with the method mentioned above.

Total RNA was extracted from blood samples using blood total RNA extraction kit (Biotek). After genotyping, the total RNAs were grouped, and every group contained a GG genotype, an AG-genotype, and an AA genotype samples randomly to avoid the instrumental and artificial errors. Real-time PCR was performed for every group according to the PrimeScript™ RT Master Mix (Perfect Real Time) (TaKaRa) and SYBR Premix Ex Taq II (TaKaRa). The primer sequences and product lengths are listed in Supplementary Table S2. The real-time PCR was run on the Thermal Cycler Dice Real Time System (TaKaRa): 95°C for 30 s, and 40 cycles of 95°C for 5 s, a 30 s annealing step at 60°C and 95°C for 15 s, 60°C for 30 s, 95°C for 15 s for dissociation.* β-actin* was used as an internal control.

The study protocol was drawn up in compliance with the principles of the Helsinki Accord and was reviewed and approved by the local Ethical Committee for both Xi'an and Kunming populations. Statement of informed consent was obtained from all participants after a full explanation of the procedure.

### 2.5. Statistical Analysis

SPSS 18.0 for Windows (PASW Statistics, SPSS, Chicago, IL, USA) was used for statistical analyses. Deviation of genotype distributions from the Hardy-Weinberg equilibrium (HWE) was checked for using the *χ*^2^-test, which was also used to assess differences in genotype and allele frequencies between study groups. Logistic regression analyses with dominant, recessive, and additive genetic models and allele contrast were used to assess the associations between* ADORA3* SNPs and CHF.

Differences in parameters related to the severity of CHF, such as left ventricular ejection fraction (LVEF), left ventricular end-diastolic diameter (LVEDD), and left ventricular end-systolic diameter (LVESD), were evaluated among patients stratified according to the three different genotypes by analysis of variance, and significant differences among pairs of means were tested using Dunnett's test. Differences in genotype distribution and allele frequency for the significant SNP according to functional New York Heart Association (NYHA) class were also analyzed by *χ*^2^-test.

Differences in luciferase activity of allelic reporter constructs were assessed by Student's *t*-test. Additionally, Dunnett's test was used to determine the differences of ADORA3 mRNA expression among the three genotype individuals, with wild type population as the reference group. The cutoff for statistical significance was set at *P* < 0.05 (two-sided).

## 3. Results

### 3.1. Case-Control Study of the SNPs with CHF

Table 1 reported the demographic and clinical characteristics of the two study populations. In the Xi'an population, there was no significant difference between the CHF patients and controls with respect to gender or age. However, the prevalence of traditional cardiovascular risk factors was significantly higher in the CHF patients in comparison with the control group (*P* < 0.05). The main etiologies of CHF are coronary artery disease (CAD) and idiopathic dilated cardiomyopathy (ICDM). Most of CHF patients show poor cardiac function with low LVEF value.

In the Kunming replication population, cases and controls were of similar age and sex ratio. The prevalence of all traditional cardiovascular risk factors was significantly higher in CHF patients in comparison with that observed in the control group (*P* < 0.01) except dyslipidemia. The main etiologies of CHF are still CAD and ICDM, and the cardiac function and LVEF for most of CHF patients are poor.

### 3.2. Distributions of Genotype and Allele between CHF Patients and Health Controls

In the Xi'an population, only the rs1544223 variant was associated with a significant predisposition to CHF under the dominant and allele contrast models. The genotype distributions of the 4 selected SNPs in the healthy controls were in accordance with the HWE. After adjustment for age, sex, and traditional cardiovascular risk factors, this SNP significantly and independently influenced the susceptibility to CHF only under a dominant model of inheritance (*P* = 0.046, OR = 1.662, 95% CI = 1.009–2.738; Table 2). In the Kunming replication population, rs1544223 was still associated with CHF risk under dominant, recessive, additive, and allele contrast models. After logistic regression, this SNP still affected CHF susceptibility under dominant, additive and allele contrast models, suggesting that this SNP was also a risk factor for Kunming CHF patients.

Genotype distributions of other three SNPs (rs1415792, rs3393, and rs35511654) and their associations with the risk of chronic heart failure under different statistical models were shown in supplementary Tables S3 and S4. Overall, no significant association was found.

**Figure 1:**
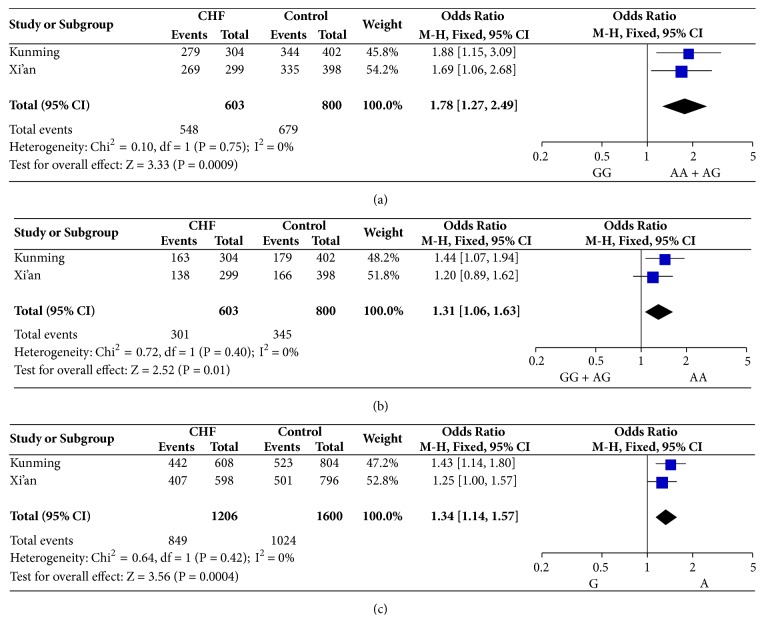
Meta-analysis of association between* ADORA3 *rs1544223 polymorphism and CHF risk under the dominant model (a), recessive model (b), and allele contrast (c), according to genotype (a) and allele (b) model.

We then performed a meta-analysis to integrate data of the two populations. The results were shown in Figure 1. We found the rs1544223 was significantly associated with CHF risk under dominant, recessive, additive, and allele contrast models when merged the data of Xi'an and Kunming together. In addition, there was no heterogeneity between the two populations, suggesting that it was valid for merging the data of two groups.

### 3.3. Association between Genotype Distribution and Allele Frequency and Clinicopathological Parameters in Patients with CHF

Then, the genotype distributions of rs1544223 were explored in relation to different CHF clinical subsets. In the Xi'an population, there was no difference in genotype distribution and allele frequency for the rs1544223 SNP according to functional NYHA class. Similarly, there was no difference in genotype distribution and allele frequency for this SNP between subsets of CHF patients (Table 3). In addition, the role of the rs1544223 was analyzed according to parameters related to the severity of CHF, such as LVEF, LVEDD, and LVESD. Carriers of the A allele appeared more likely to suffer from a higher LVEDD, although the difference did not reach significance. There was no significant difference in LVEF or LVESD between the different genotype groups (Table 4).

In the Kunming population, the similar results were found. Rs1544223 was not associated with the clinicopathological parameters of CHF patients (Tables 3 and 4).

### 3.4. SNP Effect on ADORA3 Promoter Activity

**Figure 2:**
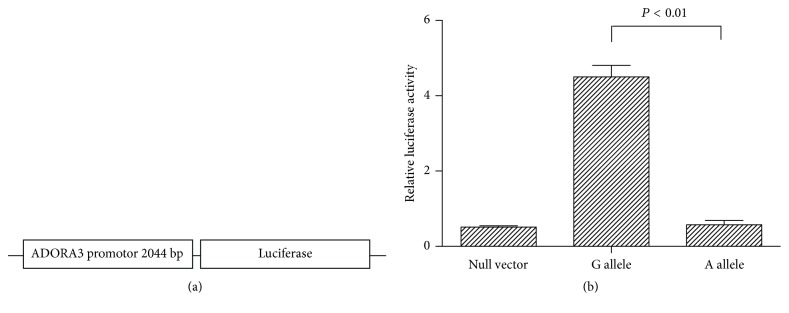
Effects of the promoter polymorphisms on the transcription activity of human ADORA3 promoter. (a) Schematic representation of reporter gene constructs that contained the ADORA3 promoter region with the rs1544223. (b) Relative luciferase activity of report gene containing different allele. Relative luciferase activity is represented as the ratio of firefly luciferase activity to renilla luciferase. Values represent mean ± SD. Every test was repeated three times. Statistical differences were evaluated with Student's *t*-test.

HEK-293 T cells were transiently transfected with plasmids containing ADORA3 gene promoter upstream of the luciferase gene. Two constructs were designed containing G or A allele at the rs1544223 SNP in the promoter. Promoter including the A allele showed a lower transcriptional activity than that with the G allele in HEK-293 T cells (*P* < 0.01; Figure 2).

### 3.5. ADORA3 mRNA Expression

**Figure 3:**
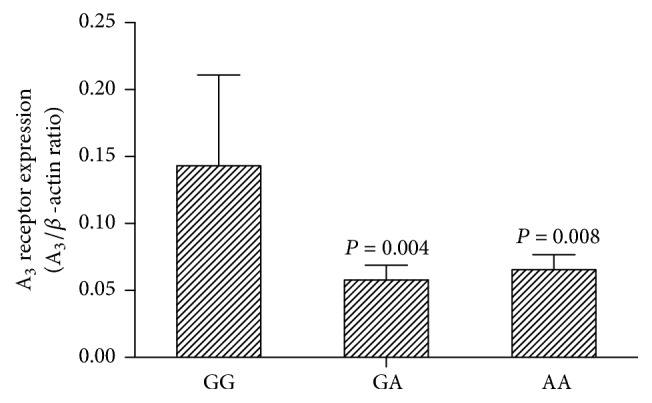
ADORA3 mRNA expression in different genotype blood samples. Data was shown as mean ± SD, *n* = 6. Statistical differences were evaluated with the Dunnett's test and the GG group was as control.

ADORA3 expression as mRNA was measured by real-time PCR in 107 blood samples from which six carried the GG genotype, 39 the GA genotype, and 62 the AA genotype at rs1544223. Six groups were therefore performed for real-time PCR. ADORA3 mRNA levels, reported as a ratio with *β*-actin mRNA, were significantly higher in GG homozygotes than subjects carrying GA (*n* = 6, 0.058 ± 0.01 versus 0.143 ± 0.068, *P* = 0.004) or AA genotypes (*n* = 6, 0.065 ± 0.01 versus 0.143 ± 0.068, *P* = 0.008; Figure 3).

## 4. Discussion

This is the first time that the effect of ADORA3 gene SNPs has been explored as a potential predisposing factor to CHF. The rs1544223 SNP was found to be a risk factor for CHF: carriers with the AA or AG genotypes had a greater risk of developing CHF than carriers with the GG genotypes, irrespective of their cardiovascular risk factors. This finding was repeated in another population. About the function of this SNP, we identified the A allele to be a low-transcript allele by using the reporter activity assay. Decreased expression of the ADORA3 gene in relation to AA/GA was also confirmed by examination of the ADORA3 mRNA expression level.

CHF is associated with increased systemic (plasma) and reduced local (myocardial) Ado levels and the final biological action of Ado in a particular organ or cell population may depend upon the relative degree of expression and signaling efficiency of individual Ado receptor subtypes. The main cardioprotective role has been allocated to the most-studied Ado receptors. For example,* A*_*1*_*R*,* A*_*2A*_*R*, and* A*_*2B*_*R *protect the heart against ischemic as cofactors of an A_1_R-mediated pathway [12, 27–30]. The least known of the Ado receptors is* ADORA3 *[22]. One study has demonstrated that activation of* ADORA3* can elicit cardioprotection by inhibiting the expression of inflammatory cytokines [12]. In addition,* ADORA3* activation can trigger sustained preconditioning effects [28]. Auchampach and colleagues have shown that selective* ADORA3* agonism during reperfusion limits damage in the canine myocardium [31]. Moreover, Maddock and colleagues have shown activation of* ADORA3* at the onset of reperfusion, thus protecting the myocardium from postischemic injury via an antiapoptotic/necrotic mechanism that was abolished in the presence of the* ADORA3* antagonist MRS 1191 [32, 33]. Yoshino et al. found ADORA3 was associated with coronary endothelial dysfunction [34]. Thus,* ADORA3* indeed mediates cardioprotective effects. In the present study, we found that one particular SNP (rs1544223) influenced the individual's predisposition for heart failure, indirectly verifying that fact.

Promoter regions containing different allele showed different transcript activity, and the mRNA level of ADORA3 among the three genotypes also showed statistical differences. All of these suggested that rs1544223 might affect the gene expression level by influencing the promoter activity. Actually, rs1544223 was located in the 5′untranslated region (5′UTR) of the A DORA3 gene. The regulation of mRNA translation initiation in eukaryotic cells is achieved mainly through a highly structured 5′UTR [35, 36]. In addition, the 5′UTR can affect the half-life of mRNA and thus indirectly regulate the stability of mRNA [37]. Based on these biological findings, we hypothesized that different genotypes of rs1544223 may lead to distinct 5′UTR sequences and further result in the disruption of the ability of this region to initiate mRNA translation and to effect mRNA stability. This might be the mechanism by which rs1544223 causes different expression level.

The role of rs1544223 on ADORA3 expression may exert some effect on the cardioprotection of this receptor. Studies in murine models have revealed that slight overexpression of* ADORA3* enhances cardioprotection with no adverse effects, but a six fold overexpression in mice could result in dilated cardiomyopathy [22, 38]. Jacobson and Gao also noted that* ADORA3* is expressed in the heart at a very low level. Hence, this receptor is either very efficiently coupled to the signaling pathways protecting the heart or its cardioprotective effects are also induced by activation of the* ADORA3* in cells outside cardiac tissues [39]. All of these suggested that the cardioprotection ability of this receptor largely depended on its expression level. In this study, individuals carrying GG genotype showed about twice higher than those carrying other genotypes, which in theory results in different capacity of cardioprotection and thus differing CHF susceptibility. But the mechanism underlying the cardioprotective influence of* ADORA3* is highly complex, and they have yet to be fully elucidated. Further related studies are needed.

The roles of rs1544223 in other diseases have been explored in many studies. Campbell et al. evaluated its effect on autism spectrum disorder, but did not find any significant associations [40]. Stamp et al. investigated the expression of* ADORA3* in rheumatoid synovial tissue, and the influence of methotrexate (MTX) exposure on this expression. They also investigated whether polymorphisms within* ADORA3* were associated with response and/or adverse effects associated with MTX. They found no association between the rs1544224 SNP and the disease activity or MTX-associated adverse effects in rheumatoid arthritis. Furthermore, since rs1544223 was found to be in incomplete linkage disequilibrium with rs1544224, there was also no association between rs1544223 and either the disease activity or MTX-associated adverse effects in rheumatoid arthritis [41]. In addition, Kim et al. studied the association between polymorphisms in the ADORA3 gene and aspirin-intolerant asthma and found that rs1544224 did not influence the risk of that disease [42]. We are not aware of any other literature on the role of rs1544223 in the heart.

Another widely studied SNP of the ADORA3 gene was rs35511654, which changes isoleucine to leucine at position 248 of the receptor; this amino acid is located in the sixth transmembrane domain of the receptor. It has been shown that nearby amino acids are involved in the recognition of agonists and antagonists as well as signal transduction and receptor activation [43]. One study focusing on the role of Ado receptors in MI has shown that rs35511654 is associated with a larger infarct. Single-proton emission computed tomography revealed an increase of ~33% in infarct size per C (minor) allele [44]. Peculis et al. examined whether this SNP is associated with coronary heart disease and found a significantly decreased frequency of the rs35511654 C allele in a group of coronary heart disease patients compared with controls [22]. In the present study we did not find this SNP in the Chinese Han population based on the HapMap database, indicating a very low minor allele frequency of this SNP in the Chinese Han population. Among our 300 CHF patients and 400 controls, we found that the A allele frequency was really very low, and so we did not study it further.

This is the first demonstration of rs1544223 being associated with susceptibility to CHF. This susceptibility suggests that genotype testing is important for identifying carriers of the risk genotypes that would increase their susceptibility to CHF. Any such people identified will be able to take the necessary protective measures, which are particularly important in those with traditional cardiovascular risk factors. The present findings did not demonstrate an influence of the* ADORA3* locus on disease severity, as no difference in allele frequency or genotype distribution was observed according to NYHA class, LVEF, LVEDD, and LVESD. These data possibly strengthen the observation that the* ADORA3* locus could be involved in protection against CHF, but not in its progression. In addition, the associations between ADORA3 SNPs and hypertension CHF, dyslipidemia CHF, diabetes CHF, or smoking habit CHF were not found, which suggested that ADORA3 activity might not affect hypertension, dyslipidemia, diabetes, or smoking habit phenotypes.

Substantial differences in geographical conditions and dietary habits exist between those residing in northern and southern China. In this study, we collected data on both northern Chinese population and southern Chinese population, reducing the bias from region or population. The main limitation of this study is that we only studied the role of rs1544223 on gene expression, and its deep function mechanism, such as its effect on transcription factors and microRNA combination, needed to be explored further.

This preliminary study has revealed an association between the ADORA3 gene and CHF thus provides new insights for investigating the contribution of Ado to heart function. The findings of the present study are intriguing because of their novelty. Nevertheless, because of the limited sample size, further studies performed in larger populations are required to better define the role of the rs1544223 SNP in influencing not only CHF susceptibility but also the severity of the disease.

## Figures and Tables

**Table 1:** Demographic and clinical characteristics of populations.

	Xi'an population			Kunming populations		
	Chronic heart failure	Controls	*P* value	Chronic heart failure	Controls	*P* value
Age (years)	61.41 ± 12.51	60.33 ± 8.56	0.2^a^	59.78 ± 7.38	59.34 ± 7.46	0.429^a^
Males/females	175/125	211/189	0.14^b^	136/168	158/244	0.15^b^
Hypertension	172 (57.33%)	122 (30.5%)	<0.001^b^	170 (55.92%)	122 (30.35%)	<0.001^b^
Dyslipidemia	84 (28%)	81 (20.25%)	0.0168^b^	70 (23.03%)	85 (21.14%)	0.55^b^
Diabetes	104 (34.67%)	67 (16.75%)	<0.001^b^	101 (33.22%)	67 (16.67%)	<0.001^b^
Smoking habit	91 (30.33%)	62 (15.5%)	<0.001^b^	91 (29.93%)	63 (15.67%)	<0.001^b^
CAD	180 (60%)	—		200 (65.79%)	—	
ICDM	76 (25.33%)	—		90 (29.61%)	—	
HC	9 (3%)	—		6 (1.97%)	—	
Other diagnose	35 (11.67%)	—		8 (2.63%)	—	
LVEF ≤ 40%	207 (69%)	—		202 (66.45%)	—	
NYHA class:						
II	94 (31.33%)	—		101 (33.22%)	—	
III	117 (39%)	—		118 (38.82%)	—	
IV	89 (29.67%)	—		85 (27.96%)	—	

CAD: coronary artery disease; ICDM: idiopathic dilated cardiomyopathy; HC: hypertensive cardiomyopathy; LVEF: left ventricular ejection fraction; NYHA: New York Heart Association class; ^a^*P* values were calculated by separate variance estimation *t*-test as variance between the two group is not neat; ^b^*P*  values were calculated from two-sided *χ*^2^-test.

**Table 2:** Genotype distribution of the rs1544223 and its associations with the risk of chronic heart failure under different contrast models.

Genotype	CHF *n* (%)	Controls *n* (%)	HWE *P*^a^	Dominant model *P*^a^; *P*^b^, OR (95% CI)	Recessive model *P*^a^; *P*^b^, OR (95% CI)	Additive model *P*^a^; *P*^b^, OR (95% CI)	Allele contrast *P*^a^; *P*^b^, OR (95% CI)
rs1544223							
(Xi'an)							
GG (W)	30 (10.03)	63 (15.83)	0.0732	0.0259; 0.046,	0.2415; 0.207,	0.0764; 0.059,	0.0471; 0.055,
AG	131 (43.81)	169 (42.46)		1.662	1.233	1.256	1.265
AA	138 (46.15)	166 (41.71)		(1.009–2.738)	(0.890–1.708)	(0.991–1.593)	(0.995–1.608)

rs1544223							
(Kunming)							
GG	25 (9.21)	58 (14.43)	0.0510	0.011; 0.048,	0.017; 0.075,	0.011; 0.025,	0.002; 0.02,
AG	116 (37.83)	165 (41.04)		1.689	1.338	1.31	1.336
AA	163 (52.96)	179 (44.53)		(1.004–2.841)	(0.971–1.843)	(1.034–1.661)	(1.046–1.706)

CHF: chronic heart failure; HWE: Hardy-Weinberg equilibrium; ^a^*P* values were calculated from two-sided *χ*^2^-test. ^b^Adjusted for age, sex, and cardiovascular risk factors (hypertension, dyslipidemia, diabetes, and smoking habit).

**Table 3:** Rs1544223 polymorphism genotype distribution and allele frequency according to NYHA and subsets of CHF patients.

Genotype	NYHA			Hypertension	Dyslipidemia	Diabetes	Smoking habit
	II	III	IV				
rs1544223 (Xi'an)							
GG	12 (12.77%)	10 (8.55%)	8 (9.09%)	15 (8.77%)	8 (9.64%)	12 (11.54%)	10 (10.99%)
AG	43 (45.74%)	50 (42.74%)	38 (43.18%)	84 (49.12%)	32 (38.55%)	41 (39.42%)	41 (45.05%)
AA	39 (41.49%)	57 (48.72%)	42 (47.73%)	72 (42.11%)	43 (51.81%)	51 (49.04%)	40 (43.96%)
Allele frequency	0.4163^a^			0.3982^b^	0.3255^b^	0.7916^b^	0.5844^b^
(*P*-value)							
Genotype distribution	0.7704^a^			0.0991^b^	0.4633^b^	0.5095^b^	0.8598^b^
(*P*-value)							
rs1544223 (Kunming)							
GG	5 (4.95%)	14 (11.86%)	6 (7.06%)	11 (6.47%)	4 (5.71%)	5 (4.95%)	5 (5.49%)
AG	36 (35.64%)	45 (38.14%)	35 (41.18%)	64 (37.65%)	24 (34.29%)	38 (37.62%)	37 (40.66%)
AA	60 (59.41%)	59 (50%)	44 (51.76%)	95 (55.88%)	42 (60%)	58 (57.43%)	49 (53.85%)
Allele frequency	0.1600^a^			0.2105^b^	0.1784^b^	0.1669^b^	0.5928^b^
(*P*-value)							
Genotype distribution	0.3193^a^			0.3992^b^	0.4180^b^	0.3026^b^	0.4989^b^
(*P*-value)							

NYHA: New York Heart Association class; ^a^*χ*^2^-test -test: allele frequency and genotype distribution among the three groups; ^b^*χ*^2^-test -test: allele frequency and genotype distribution versus controls.

**Table 4:** Parameters related to the severity of CHF in different genotype groups.

Genotype	LVEF	LVEDD	LVESD
	mean value	mean value	mean value
rs1544223 (Xi'an)			
GG	40.73 ± 13.51	64.30 ± 10.30	52.07 ± 11.73
AG	41.94 ± 13.83	64.61 ± 11.56	51.41 ± 13.50
AA	39.88 ± 11.89	65.70 ± 10.78	53.46 ± 12.19
*P* ^a^	0.428	0.664	0.417

rs1544223 (Kunming)			
GG	34.88 ± 12.99	67.36 ± 8.91	55.88 ± 11.03
AG	41.35 ± 15.41	65.25 ± 11.38	51.89 ± 14.05
AA	40.26 ± 13.07	64.56 ± 10.89	51.72 ± 12.60
*P* ^a^	0.113	0.477	0.325

LVEF: left ventricular ejection fraction; LVEDD: left-ventricle end-diastolic diameter; LVESD: left-ventricle end-systolic diameter. *Note*. Multiple comparisons among pairs of means were also tested using Dunnett's test and no significance was found; ^a^*P* values were calculated from two-sided Analysis of Variance.

## Data Availability

Readers can get the original data by contacting the author.

## Supplementary Materials

Supplementary Materials Supplementary Table S1: primers used for SNP genotyping. Supplementary Table S2: primer lists of real-time PCR. Supplementary Table S3: genotype distribution of the rs1415792, rs3393, and rs35511654 and their associations with the risk of chronic heart failure under different contrast models. Supplementary Table S4: logistic regression analysis for rs1415792, rs3393, and rs35511654 under a dominant, recessive, and additive genetic model adjusted for age, sex, and cardiovascular risk factors.
