# Supplementary material for: The Polymorphism in ADORA3 Decreases Transcriptional Activity and Influences the Chronic Heart Failure Risk in the Chinese
Source: Biomed Res Int. 2018 May 31;2018:4969385. doi: 10.1155/2018/4969385 (PMC6000890; doi:10.1155/2018/4969385)
Supplement: Supplementary Materials — Supplementary Table S1: primers used for SNP genotyping. Supplementary Table S2: primer lists of real-time PCR. Supplementary Table S3: genotype distribution of the rs1415792, rs3393, and rs35511654 and their associations with the risk of chronic heart failure under different contrast models. Supplementary Table S4: logistic regression analysis for rs1415792, rs3393, and rs35511654 under a dominant, recessive, and additive genetic model adjusted for age, sex, and cardiovascular risk factors. [file 4969385.f1.pdf]

The polymorphism in *ADORA3* decreases transcriptional activity and influences the chronic heart failure risk in Chinese

Hai-Rong He (M.M.) [1,a], Yuan-Jie Li (M.D.; Ph.D.)[2,a], Gong-Hao He (M.D.; Ph.D.)[3], Hua Qiang (Ph.D.)[4], Ya-Jing Zhai (B.S.)[5], Mao Ma (Ph.D.)[6], Ya-Jun Wang (B.S.)[6], Jun Lyu (M.D.; Ph.D.)[1,*]

[1]*Clinical Research Center, The First Affiliated Hospital of Xi'an Jiaotong University, Xi'an, 710061, China*

[2]*Department of Human Anatomy, Histology and Embryology, Medical College, Xi'an Jiaotong University, Xi'an, 710061, China.*

[3]*Department of Pharmacy, Kunming General Hospital of Chengdu Military Region, Kunming 650032, China.*

[4]*Department of Cardiovascular Medicine, The First Affiliated Hospital of Xi'an Jiaotong University, Xi'an, 710061, China.*

[5]*Department of Pharmacy, The First Affiliated Hospital of Xi'an Jiaotong University, Xi'an, 710061, China.*

[6]*Physical Examination Department, The First Affiliated Hospital of Xi'an Jiaotong University, Xi'an, 710061, China*

List of Supplementary Material for the article

1. Supplementary Table S1: Primers used for SNP genotyping.
2. Supplementary Table S2: Primer lists of real-time PCR.
3. Supplementary Table S3: Genotype distribution of the rs1415792, rs3393 and rs35511654 and their associations with the risk of chronic heart failure under different contrast models.
4. Supplementary Table S4: Logistic regression analysis for rs1415792, rs3393 and rs35511654 under a dominant, recessive, and additive genetic model adjusted for age, sex, and cardiovascular risk factors.

Supplementary Table S1. Primers used for SNP genotyping.

SNP_ID	Allele	1st-PCRP	2nd-PCRP	UEP_SEQ
rs1415792	T/C	ACGTTGGATGGCAGGTTCTCTGCTCAATTC	ACGTTGGATGGACTTACTGACAAAAGGCTC	TGGTGGTGACTCATTTCCAT
rs1544223	G/A	ACGTTGGATGAGAGGACAGTGCAGGATCAG	ACGTTGGATGTTTGCTAAGCTGGCAGAAAG	gaTGGCAGAAAGATTGCATA
rs3393	G/A	ACGTTGGATGGTGGAATTGAGCAGAGAACC	ACGTTGGATGTTCAGCAGTTTAAGTCCCCC	TTCTGTTCCCAACATCT
rs35511654	T/G	ACGTTGGATGAGCACAAGCTGTGGTACCTC	ACGTTGGATGTGTTTGCTCTGTCATGGCTG	ctcttTCATGGCTGCCTTTATCT

Supplementary Table S2. Primer lists of real-time PCR.

Gene	Forward primer (5'→3')	Reverse primer (5'→3')	Product size (bp)
ADORA3	AGCAAAGCGTCAACTCGTG	ATAAGGAGGCAAACGGGAGA	112
β-actin	TGACGTGGACATCCGCAAAG	CTGGAAGGTGGACAGCGAGG	205

Supplementary Table S3. Genotype distribution of the rs1415792, rs3393 and rs35511654 and their associations with the risk of chronic heart failure under different contrast models.

Population	Genotype	CHF n (%)	Controls n (%)	HWE *P*[a]	Dominant model *P*[a]	Recessive model *P*[a]	Additive model *P*[a]	Allele contrast *P*[a]
Xi' an	rs1415792							
	TT (W)	18 (6)	14 (3.5)	0.043	0.117	0.691	0.207	0.826
	CT	105 (35)	156 (39)					
	CC	177 (59)	230 (57.5)					
	rs3393							
	GG (W)	97 (32.33)	146 (36.68)	0.063	0.232	0.996	0.440	0.416
	GA	145 (48.33)	175 (43.97)					
	AA	58 (19.33)	77 (19.35)					
	rs35511654							
	TT (W)	298 (99.33)	398 (99.5)	0.96	0.828[b]	NA	NA	0.828
	GT	2 (0.67)	2 (0.5)					
	GG	0 (0)	0 (0)					
Kunming	rs1415792							
	TT (W)	15 (4.98)	28 (7)	0.313	0.271	0.526	0.519	0.338
	CT	105 (34.88)	141 (35.25)					
	CC	181 (60.13)	231 (57.75)					
	rs3393							
	GG (W)	78 (25.74)	130 (32.42)	0.076	0.055	0.301	0.147	0.063
	GA	150 (49.5)	185 (46.13)					
	AA	75 (24.75)	86 (21.45)					
	rs35511654							
	TT (W)	302 (99.67)	400 (99.75)	NA	0.606[b]	NA	NA	0.606
	GT	1 (0.33)	1 (0.25)					
	GG	0 (0)	0 (0)					

Abbreviations: CHF, chronic heart failure; HWE, Hardy-Weinberg equilibrium; NA, not available.

[a] *P* values was calculated from two-sided χ^2-test.

[b] *P* values was calculated from two-sided continuity-adjusted $\chi 2$-test.

Supplementary Table S4. Logistic regression analysis for rs1415792, rs3393 and rs35511654 under a dominant, recessive, and additive genetic model adjusted for age, sex, and cardiovascular risk factors.

		Xi'an		Kunming	
SNPs	Contrast models	Odds ratio (95% CI)	*P*[a]	Odds ratio (95% CI)	*P*[a]
rs1415792	Dominant model	0.488 (0.227-1.050)	0.067	1.157 (0.587-2.281)	0.674
	Recessive model	0.931 (0.671-1.290)	0.666	0.997 (0.720-1.381)	0.987
	Additive model	0.865 (0.656-1.140)	0.303	1.021 (0.784-1.329)	0.879
	Allele contrast	0.872 (0.667-1.140)	0.317	1.021 (1.782-1.333)	0.877
rs3393	Dominant model	1.201 (0.854-1.688)	0.292	1.259 (0.883-1.794)	0.203
	Recessive model	1.091 (0.728-1.636)	0.674	1.120 (0.767-1.637)	0.556
	Additive model	1.112 (0.889-1.390)	0.354	1.138 (0.912-1.421)	0.252
	Allele contrast	1.118 (0.889-1.405)	0.342	1.144 (0.913-1.435)	0.242
rs35511654	Dominant model	1.531 (0.205-11.465)	0.678	1.364 (0.078-23.869)	0.832
	Recessive model	NA	NA	NA	NA
	Additive model	NA	NA	NA	NA
	Allele contrast	1.526 (0.205-11.336)	0.680	1.361 (0.078-23.628)	0.832

Abbreviations: CI, confidence interval; NA, not available.

[a]Adjusted for age, sex, and cardiovascular risk factors(hypertension, dyslipidemia, diabetes and smoking habit).
